# Got ACTs? Availability, price, market share and provider knowledge of anti-malarial medicines in public and private sector outlets in six malaria-endemic countries

**DOI:** 10.1186/1475-2875-10-326

**Published:** 2011-10-31

**Authors:** Kathryn A O'Connell, Hellen Gatakaa, Stephen Poyer, Julius Njogu, Illah Evance, Erik Munroe, Tsione Solomon, Catherine Goodman, Kara Hanson, Cyprien Zinsou, Louis Akulayi, Jacky Raharinjatovo, Ekundayo Arogundade, Peter Buyungo, Felton Mpasela, Chérifatou Bello Adjibabi, Jean Angbalu Agbango, Benjamin Fanomezana Ramarosandratana, Babajide Coker, Denis Rubahika, Busiku Hamainza, Steven Chapman, Tanya Shewchuk, Desmond Chavasse

**Affiliations:** 1 Population Services International, Malaria & Child Survival Department, P.O. Box 43640, Nairobi, Kenya; 2 Department of Global Health and Development, London School of Hygiene and Tropical Medicine, 15-17 Tavistock Place, London WC1H 9SH, UK; 3 Association Béninoise pour le Marketing Social/PSI, B.P. 08-0876, Tri Postal, Cotonou, Benin; 4 Association de Santé Familiale, 4630 Avenue de la Science, Immeuble USTC, Bloc C, Gombe, Kinshasa, Democratic Republic of Congo; 5 PSI/Madagascar, Immeuble- FIARO, Rue Jules Ranaivo, Escalier-D, 2eme Etage, BP 7748, Antananarivo 101, Madagascar; 6 Society for Family Health, 8 Port Harcourt Crescent, Area 11 Garki Abuja, Nigeria; 7 PACE, Plot 2 Ibis Vale, P.O. Box 27659, Kololo, Kampala, Uganda; 8 Society for Family Health, Plot No. 549, Ridgeway, P.O. Box 50770, Lusaka, Zambia; 9 Ministère de la Santé, Programme National de Lutte contre le Paludisme (PNLP), Akpakpa, Cotonou, Bénin; 10 National Malaria Control Programme, 1, Avenue du Tourisme, Ngaliema, Kinshasa, Democratic Republic of Congo; 11 Ministre de la Santé Publique, Centre National de Lutte contre le Paludisme (CNLP), Androhibe Antananarivo, 101, Madagascar; 12 National Malaria Control Programme, Abia House, Near Adamawa Plaza, First Avenue, Central District Area, Postal Code 900001, Federal Capital Territory, Abuja, Nigeria; 13 Ministry of Health, Malaria Control Programme, Lourdel Rd Wandegeya, Kampala, Uganda; 14 National Malaria Control Center, P.O Box 32509, Lusaka, Zambia; 15 Population Services International, 1120 19th Street N.W., 20036, Washington D.C., USA

## Abstract

**Background:**

Artemisinin-based combination therapy (ACT) is the first-line malaria treatment throughout most of the malaria-endemic world. Data on ACT availability, price and market share are needed to provide a firm evidence base from which to assess the current situation concerning quality-assured ACT supply. This paper presents supply side data from *ACTwatch *outlet surveys in Benin, the Democratic Republic of Congo (DRC), Madagascar, Nigeria, Uganda and Zambia.

**Methods:**

Between March 2009 and June 2010, nationally representative surveys of outlets providing anti-malarials to consumers were conducted. A census of all outlets with the potential to provide anti-malarials was conducted in clusters sampled randomly.

**Results:**

28,263 outlets were censused, 51,158 anti-malarials were audited, and 9,118 providers interviewed. The proportion of public health facilities with at least one first-line quality-assured ACT in stock ranged between 43% and 85%. Among private sector outlets stocking at least one anti-malarial, non-artemisinin therapies, such as chloroquine and sulphadoxine-pyrimethamine, were widely available (> 95% of outlets) as compared to first-line quality-assured ACT (< 25%). In the public/not-for-profit sector, first-line quality-assured ACT was available for free in all countries except Benin and the DRC (US$1.29 [Inter Quartile Range (IQR): $1.29-$1.29] and $0.52[IQR: $0.00-$1.29] per adult equivalent dose respectively). In the private sector, first-line quality-assured ACT was 5-24 times more expensive than non-artemisinin therapies. The exception was Madagascar where, due to national social marketing of subsidized ACT, the price of first-line quality-assured ACT ($0.14 [IQR: $0.10, $0.57]) was significantly lower than the most popular treatment (chloroquine, $0.36 [IQR: $0.36, $0.36]). Quality-assured ACT accounted for less than 25% of total anti-malarial volumes; private-sector quality-assured ACT volumes represented less than 6% of the total market share. Most anti-malarials were distributed through the private sector, but often comprised non-artemisinin therapies, and in the DRC and Nigeria, oral artemisinin monotherapies. Provider knowledge of the first-line treatment was significantly lower in the private sector than in the public/not-for-profit sector.

**Conclusions:**

These standardized, nationally representative results demonstrate the typically low availability, low market share and high prices of ACT, in the private sector where most anti-malarials are accessed, with some exceptions. The results confirm that there is substantial room to improve availability and affordability of ACT treatment in the surveyed countries. The data will also be useful for monitoring the impact of interventions such as the Affordable Medicines Facility for malaria.

## Background

In 2000, African Heads of State agreed in Abuja that by 2010 60% of people with malaria would be able to access "*affordable and appropriate treatment within 24 hours of the onset of symptoms*" [1]. This target was increased to 80% in 2005 [2]. Artemisinin-based Combination Therapy (ACT) is the first-line malaria treatment throughout most of the malaria-endemic world. Although public sector procurement of ACT has increased sharply in recent years, it was estimated that in 2009 less than 15% of children under five years of age received ACT when they had fever in 11 of 13 African countries, and only 32% received anti-malarials of any kind [3]. Furthermore, the availability and use of oral artemisinin monotherapy [4,5] is worrying given the risk of development of artemisinin resistance [6] and WHO's recommendation to countries to ban the importation and manufacturing of these drugs.

A variety of country-specific studies have raised important concerns about ACT supply that may explain these low levels of ACT use. Studies have generally found low availability of ACT and higher availability of monotherapies in both the public and private sector [7-9]. While ACT is typically free-of-charge or subsidized in the public and not-for-profit sector, in the private sector ACT is between ten and twenty times more expensive than non-artemisnin therapies, such as chloroquine and sulphadoxine-pyrimethamine (SP) [8,10-12]. Provider knowledge that ACT is the first-line treatment for malaria is also low, particularly in the private sector [13-16].

Research addressing questions on the anti-malarial market is typically limited in geographical scope and utilizes a variety of different methods to ascertain price and availability. Data are typically collected in a few purposively selected geographical areas within one country, and at times include only certain outlet types or sectors. Studies are not typically designed with sufficient power to detect differences between sectors. With a few exceptions [14,17-20], there is little evidence on the market share of anti-malarial medicines sold through the private retail sector, where most malaria treatment is sought.

The available data, therefore, fail to provide a firm evidence base from which to assess the current situation concerning ACT supply across countries, or to help evaluate the impact of important initiatives to improve this, such as the Affordable Medicines Facility for Malaria (AMFm), which provides heavily subsidized, quality assured, ACT to pilot countries [21]. Standardized information on availability, price and market share of ACT and other anti-malarials in the public/not-for-profit and private sectors will inform such interventions and help gauge their relative success, guiding policy makers and programmers at the national and international levels.

To help address some of these gaps, Population Services International (PSI) in partnership with the London School of Hygiene and Tropical Medicine (LSHTM) launched a five-year multi-country research project in 2008 called *ACTwatch *to monitor anti-malarial supply and demand in seven malaria endemic countries in Africa and Asia. This involves multiple surveys of households and anti-malarial outlets, and a systematic analysis of the anti-malarial supply chain in Benin, Cambodia, the Democratic Republic of Congo (DRC), Madagascar, Nigeria, Uganda and Zambia [22].

This paper presents the results from *ACTwatch *outlet surveys carried out between March 2009 and June 2010 in the six sub-Saharan countries. An ACT was the first-line anti-malarial in all these countries at the time of the study, with artemether-lumefantrine and/or artesunate-amodiaquine the recommended combination. All countries recommended quinine for treatment of severe malaria, and SP for intermittent preventive treatment in pregnancy.

## Methods

### Design & sampling

A nationally representative sample of outlets providing anti-malarials to consumers was selected. Each country was first stratified using information on country size, malaria endemicity, geopolitical areas, or urbanization. Benin was designated as a single stratum; Madagascar, Uganda, and Zambia were divided into two strata; the DRC into four and Nigeria into six [22]. Stratification was agreed in consultation with national authorities including the national malaria control programme and the national drug regulatory authority.

Following stratification, a one-stage probability-proportional-to-size (PPS) cluster design was used to select clusters within each stratum, with cluster population serving as the measure of size. The primary sampling unit, or cluster, selected was an administrative unit with 10,000 to 15,000 inhabitants. The proportion of outlets with any ACT in stock was used as a key indicator for sample size calculation, conservatively estimated at 40% for each country. A minimum of 290 outlets with anti-malarials in stock was needed to detect a 20% increase in this indicator, at 80% power, setting the level of significance at 5% and adjusting for an estimated design effect of 3. It was estimated that the selection of 19 clusters per stratum would provide at least this number of outlets stocking anti-malarials.

Within each selected cluster all outlet types with the potential to provide anti-malarials to consumers were sampled, with the identification of these outlet types based on available literature and local knowledge. The main types of outlets sampled included public and not-for profit health facilities, private health facilities, pharmacies, drug stores and grocery stores/kiosks. In addition, a range of other outlet types were considered relevant in specific countries, for example, market stalls in Benin, *gargotes *in Madagascar, itinerant vendors in Nigeria and Benin, and community health workers in Nigeria, Uganda and Madagascar.

As public health facilities and pharmacies are important providers of anti-malarials but are relatively uncommon, over-sampling was conducted for these outlet types. This booster sample was obtained by including public health facilities and pharmacies in the larger administrative area from which a given cluster was selected. For example, if the cluster was defined as the sub-district, the booster sample covered all public health facilities and pharmacies in the whole district within which the sub-district was located.

### Training and fieldwork

The outlet surveys were conducted during the months of peak malaria transmission in each country. The survey data were collected by teams of between 21 and 76 enumerators with at least 12 years of education who were fluent in the local language. Interviewer training was provided over six days, focusing on outlet identification, informed consent procedures, and the procedures for completing the questionnaire.

Field workers were provided with a list of selected clusters and maps that illustrated their administrative boundaries, in addition to lists of public health facilities and pharmacies obtained from relevant authorities. Snowball sampling was also used by field workers to identify facilities that were not on official lists. In each selected cluster, fieldworkers conducted a census of all outlets that had the potential to provide anti-malarials. For each outlet that was identified during the census, the outlet type and location were noted, along with its longitude and latitude coordinates (obtained via hand-held global positioning units). A fieldworker then approached the outlet's main seller or provider and invited him or her to participate in the study. Providers who agreed to participate were asked two screening questions to determine whether (a) the outlet had stocked anti-malarials within the previous three months, and (b) anti-malarials were available on the day of the survey. All providers answering "yes" to at least one of these questions were deemed eligible for inclusion in the survey and the questionnaire was administered to these outlets. An interview with the staff member who was most likely to sell or prescribe medications was conducted.

The interview was carried out in the appropriate local language. Questionnaires were pre-tested and validated in each country before data collection.

The questionnaire consisted of two parts. A drug audit collected information on all anti-malarials found at the time of the interview, including information on brand and generic names, dose, manufacturer, amount sold in the last seven days, and retail price. The provider questionnaire collected information on provider demographics, anti-malarial treatment knowledge, anti-malarial storage conditions, stock-outs, and licensing.

Data collection lasted from one to five months in each country and took place between March 2009 and June 2010 (Benin, April 28^th ^- May 13^th ^2009; the DRC, August 10^th ^- October 27^th ^2009; Madagascar, April 27^th ^- June 21^st ^2010; Nigeria August 4^th ^- September 16^th ^2009; Uganda, March 16^th ^- April 7^th ^2009; Zambia, April 14^th ^- July 3^rd ^2009).

During data collection, approximately 80% of all questionnaires were reviewed by the team supervisor and 15-20% of all outlets were revisited by a supervisor or/and quality controller for quality control checks.

### Data analysis

Double data entry was conducted using Microsoft Access (Microsoft Corporation, Redmond, Washington, USA) with built-in range and consistency checks. Entered data were triangulated with questionnaires, field supervision notes and daily activity logs filled by each interviewer in the field. Data were analysed using Stata 11.0 (StataCorp College Station, TX).

Stata survey settings were used to account for the stratified and clustered sampling strategy. Results were adjusted by sampling weights. Sampling weights were based on the inverse of the probability of selection to allow for the differences in strata and cluster sizes and the oversampling of public health facilities and pharmacies. Distribution of the outlets was assumed to be proportional to population size. Data analysis included descriptive summaries; between-group proportions were compared using chi-square analysis and median prices compared using the Wilcoxon rank sum test.

For the presentation of results, outlets were grouped into two categories: 1) the public and not-for-profit sector, and 2) the private sector. The former included public health facilities, facilities supported by NGOs, mission hospitals and/or community health workers. The private sector included all for-profit outlets. For the purpose of analysis, anti-malarials were classified into ACT, non-artemisinin therapies (such as chloroquine and SP) and artemisinin monotherapies. ACT was classified as two mutually exclusive categories: quality-assured (appearing on the WHO list of approved formulations of ACT or the UNICEF procurement records) and non-quality assured. Quality-assured ACT was further divided into 1) quality-assured first-line ACT and 2) quality-assured non first-line ACT (see Table 1). Artemisinin monotherapies were further divided into oral and non-oral artemisinin monotherapies. Data were not collected on drugs intended solely for malaria chemoprophylaxis as the focus was on drugs for treatment. The antibiotic cotrimoxazole was also excluded as, although it has anti-malarial properties, it is very rarely used as a malaria treatment.

**Table 1:** Description of Quality-Assured ACT classifications

	First-line quality-assured ACTs:	Non first-line quality-assured ACTs:
Definitions	Government recommended first-line treatments (regardless of strength) for uncomplicated malaria that appear on the WHO list of approved ACTs or the UNICEF procurement records. Brands on these lists and audited in each country were:	ACTs that are not the government's recommended first-line treatment for uncomplicated malaria, but which appear on the WHO list of approved ACTs or the UNICEF procurement records. Brands on these lists and audited in each country were:

**Benin**	Artefan 20/120; Coartem; Lumartem 20/120; Lumet 20/120	Coarsucam; Winthrop

**DRC**	Arsuamoon; Artesunate & Amodiaquine from Ipca; Artesunate & Amodiaquine from Cipla; Falcimon Kit; Serenadose; Coarsucam; Winthrop	Artefan 20/120; Coartem 20/120; Lumartem 20/120

**Madagascar**	Arsuamoon; Artesunate & Amodiaquine from Ipca; Actipal 50/153; Winthrop; Coarsucam; Falcimon kit; Larimal	Artefan 20/120; Coartem 20/120; Coartem D 20/120; Lumartem 20/120

**Nigeria**	Coartem 20/120; Coartem D 20/120; Lumerax 20/120 *Arsuamoon; *Coarsucam; *Larimal	Artecospe

**Uganda**	Coartem; Artefan 20/120; Lumartem 20/120	Larimal, Falcimon kit

**Zambia**	Artefan 20/120; Coartem 20/120; Lumartem 20/120; Lumerax; Lumet 20/120	Arsuamoon; Artecospe

*Alternate first-line treatment in Nigeria

### Availability

Availability was calculated using two methods. For each country, availability of anti-malarials was measured as the proportion of outlets with at least one anti-malarial in stock, among all censused outlets. Within each country, the availability of specific anti-malarial categories, restricted to those outlets that had anti-malarials in stock, was also calculated. Thus, for example, the availability of ACT was measured as the proportion of outlets stocking ACT, among all outlets with at least one anti-malarial in stock.

In a supplementary analysis, the availability in public health facilities was also measured as the proportion of facilities with different categories of anti-malarials in stock, among all censused public health facilities. This operationalized the expectation that public health facilities should stock anti-malarials.

### Volumes and price

The volume sold/distributed and retail price of the anti-malarials recorded in the drug audit were standardized using the adult equivalent treatment dose (AETD) to allow meaningful comparisons between anti-malarials with different treatment courses. For the five countries that conducted surveys in 2009, prices were converted to US dollars using their average annual exchange rate [23]. For Madagascar (where data were collected in 2010), local currency prices were adjusted to 2009 prices using the Madagascar consumer price index [24], and then converted to US dollars using the 2009 average exchange rate.

Prices per AETD are presented for the three anti-malarial categories believed to be most pertinent to policy level decisions; first-line quality-assured ACT, the most popular anti-malarial based on volume (defined by generic name), and oral artemisinin monotherapy.

Price measures include tablet anti-malarials only as this is the most common formulation. The price of non-tablet formulations, such as powders for reconstitution, suspensions, suppositories and syrups, was excluded. The price distributions for these non-tablet formulations tend to be different from those for tablets, with much higher medians, implying that it could be misleading to use one measure of central tendency for all formulations. However, measures of volume include all dosage forms to provide a complete assessment of anti-malarial market shares.

### Ethical approval

The study was approved by the local ethical review boards: Benin Ministry of Health, Authorization for Data Collection Number 3989/MS/DC/SGM/DRS/SCI/SA, 14^th ^July 2008; Cambodia, Ministry of Health, National Ethics Review Committee, Reference Number 109 NECNR, 7^th ^November 2008; Democratic Republic of Congo, Ministerie de L'Enarignement Superior at Universitaire, School of Public Health, Reference Number ESP/CF/020/2008, 11^th ^June 2008; Madagascar, Ministry of Health du Planning Familial et de la Protection Sociale, Reference Number 206SANPFPS, 16^th ^June 2008; Nigeria; Federal Department of Ministry of Health, Department of Public Health, Reference Number MH/1158/5/137, 20^th ^October 2008; Uganda, Makerere University, Faculty of Medicine, Reference Number 2008-057, 1^st ^September 2008; and Zambia, University of Zambia, Biomedical Ethical Research Committee, Reference Number 014-08-08, 2^nd ^October 2008.

## Results

A total of 28,263 outlets across six countries were approached to participate in the surveys. In total, 348 outlets were closed down permanently, 665 outlets were not open, 299 eligible providers were not available for interview, 395 providers refused to participate and 301 outlets were excluded for other reasons. As a result, 26,255 outlets were screened for stocking anti-malarials. Of these, 9,118 outlets met the screening criteria and were administered the questionnaire (8,383 outlets were found to have at least one anti-malarial in stock and 735 outlets had no anti-malarials in stock at the time but reported having stocked anti-malarials in the past three months).

Among outlets stocking anti-malarials on the day of survey, 51,158 individual anti-malarial products were audited (66% tablets). The proportion of tablet formations in the audit varied by country (Benin, 73%, n=5, 232; the DRC, 58%, n=11, 939; Madagascar, 87%, n=5,579; Nigeria, n=61%; n=20,841; Uganda, 63%, n=5,784; and Zambia, 82%, n=1,783).

### Availability

Table 2 shows the availability of anti-malarials as the proportion of outlets with at least one anti-malarial in stock, among all censused outlets. The proportion of outlets with any anti-malarials in stock at the time of the interview varied by outlet type. Overall, stocking rates in the public/not-for-profit sector were generally high (~90%) with the exception of Madagascar (40%) and Uganda (70%). Both these countries had large numbers of CHWs included in the census, with less than half of these providers having anti-malarials available (27% and 40% respectively). In public health facilities, stocking rates were greater than 90% across all countries.

**Table 2:** Proportion ^(N) ^of outlets censused with at least one anti-malarial in stock on the day of interview, by sector, outlet type and country¹

	Public Sector/Not-for-Profit				Private Sector							Total
	
	Public Health Facility	Community Health Worker	Private not-for-profit Health Facility	Total Public/Not-for-Profit	Private Health Facility	Pharmacy	Drug Store	Grocery Store	Other Outlet Types		Total Private	All
											
									Shop/Kiosk/Bar/Market Stall	Itinerant Provider		
Benin	95.4 ^(182)^	-	91.2 ^(47)^	94.0 ^(229)^	84.2 ^(118)^	96.7 ^(118)^	-	30.5 ^(433)^	34.4 ^(691)^	42.7 ^(81)^	36.3 ^(1, 441)^	39.0 ^(1, 670)^
DRC	96.8 ^(111)^	-	97.2 ^(33)^	96.9 ^(144)^	75.7 ^(204)^	100.0 ^(33)^	96.5 ^(1, 089)^	-	1.7 ^(2, 245)^	-	24.9 ^(3, 571)^	28.4 ^(3, 715)^
Madagascar	96.8 ^(531)^	26.8 ^(226)^	80.6 ^(7)^	40.4 ^(764)^	87.6 ^(87)^	99.6 ^(69)^	97.4 ^(263)^	33.1 ^(5, 056)^	1.1 ^(530)^	-	33.9 ^(6, 005)^	35.0 ^(6, 769)^
Nigeria	91.8 ^(255)^	80.0 ^(19)^	98.7 ^(11)^	89.2 ^(285)^	91.4 ^(405)^	99.5 ^(409)^	95.6 ^(1, 031)^	3.8 ^(2, 141)^	2.5 ^(1, 164)^	70.2 ^(21)^	25.7 ^(5, 171)^	26.6 ^(5, 456)^
Uganda	95.4 ^(525)^	39.8 ^(90)^	88.6 ^(11)^	69.6 ^(626)^	96.2 ^(208)^	99.3 ^(97)^	96.4 ^(398)^	0.4 ^(3, 747)^	0.0 ^(191)^	-	13.9 ^(4.641)^	17.0 ^(5, 267)^
Zambia	97.4 ^(165)^	-	100.0 ^(16)^	97.8 ^(181)^	92.3 ^(34)^	100.0 ^(50)^	76.5 ^(165)^	3.7 ^(1, 946)^	0.4 ^(997)^	14.7 ^(5)^	6.3 ^(3, 197)^	9.5 ^(3, 378)^

¹In Benin the drug store category was deemed redundant as, strictly, there were no unregulated private-sector medicine vendors operating from formal structures (such as permanent buildings). In Benin, such medicine selling outlets should be registered and appear on the list of pharmacies. Unregulated vendors do operate however, often in markets, and were thus captured by the market stall and market shop classifications. In the DRC, grocery stores, as defined for all other countries, were very rare. In the DRC and Zambia, community health workers were not targeted for inclusion due to difficulties in ascertaining their presence in the clusters. In Benin, community health workers were not formally included in the Ministry of Health structure at the time of data collection [25]. It is expected that community health workers will be included in later survey rounds, as their numbers increase and as the Ministry of Health makes moves to include them explicitly in national policy [26].

In the private sector, stocking of anti-malarials was lower and varied by outlet type and country, ranging from 6% in Zambia to 36% in Benin. Private health facilities, pharmacies and drug stores were more likely to stock anti-malarials than grocery stores and other outlets, where stocking rates were generally less than 5%. Exceptions were grocery stores in Benin and Madagascar and market stalls in Benin, with around one in three of these outlets stocking anti-malarials.

**Figure 1:**
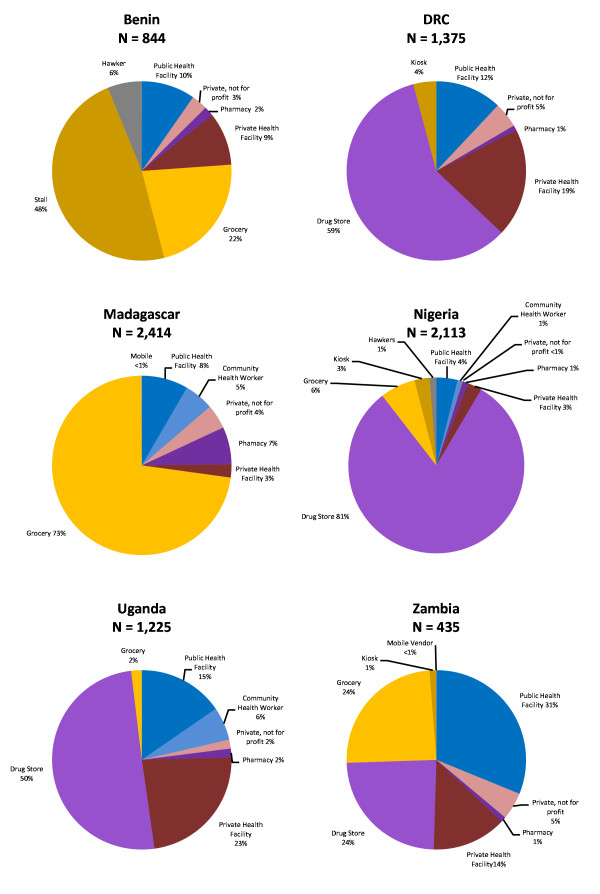
**Outlets stocking anti-malarials, by outlet type**.

Figure 1 shows the relative distribution of all outlets that had at least one anti-malarial in stock, by country. Results show the diversity of distribution of outlet types stocking anti-malarials. For example, public health facilities varied from 4% of all outlets stocking anti-malarials in Nigeria to 31% in Zambia. In Madagascar, grocery stores constituted 73% of all outlets stocking anti-malarials, as compared to 2% in Uganda. Drug stores were the most common type of outlet stocking anti-malarials in Nigeria, Uganda and the DRC; groceries and stalls were most common in Madagascar and Benin respectively; and public health facilities were the most common outlets stocking anti-malarials in Zambia.

Among outlets stocking at least one anti-malarial (Table 3), non-artemisinin therapies were generally the most commonly stocked category of anti-malarial in both the public/not-for-profit sector and private sector. A significant exception to this was found in Madagascar's public/not-for-profit sector, which was more likely to stock first-line quality-assured ACT (92%) than non-artemisinin therapies (36%). In the private sector, stocking rates of first-line quality-assured ACT were less than 25% across all countries, and for most countries significantly lower than the public/not-for-profit sector (p < .05) (Table 3). Stocking rates of oral artemisinin monotherapies were generally low, though exceptions included private sector outlets in the DRC (41%) and Nigeria (45%).

**Table 3:** Outlets with specific anti-malarial categories as a percentage of outlets with at least one anti-malarial in stock, by sector¹

	Benin		DRC		Madagascar		Nigeria		Uganda		Zambia	
	
	Public/Not- for-Profit	Private	Public/Not- for-Profit	Private	Public/Not- for-Profit	Private	Public/Not- for-Profit	Private	Public/Not- for-Profit	Private	Public/Not- for-Profit	Private
	N=212	N=632	N=134	N=1, 240	N=560	N=1, 854	N=249	N=1, 864	N=534	N=691	N=176	N=259
Any ACT	71.4 ^a^	6.4 ^b^	84.4 ^a^	52.7 ^b^	92.2 ^a^	8.4 ^b^	60.6 ^a^	36.7 ^a^	81.5 ^a^	20.1 ^b^	82.2 ^a^	19.3 ^b^
First-line quality-assured ACT	66.4 ^a^	5.7 ^b^	75.8 ^a^	24.5 ^b^	91.9 ^a^	7.9 ^b^	49.2 ^a^	25.3 ^b^	73.4 ^a^	7.8 ^b^	81.5 ^a^	16.3 ^b^
Quality-assured ACT	66.4 ^a^	5.9 ^b^	78.7 ^a^	29.0 ^b^	91.9 ^a^	8.1 ^b^	49.2 ^a^	26.1 ^a^	73.4 ^a^	8.1 ^b^	81.5 ^a^	16.3 ^b^
Non-Quality-assured ACT	5.1 ^a^	0.5 ^b^	5.6 ^a^	23.6 ^b^	0.3 ^a^	0.3 ^a^	11.5 ^a^	10.5 ^a^	9.3 ^a^	15.2 ^a^	0.8 ^a^	2.9 ^b^

Any non-artemisinin therapy	96.7 ^a^	99.7 ^b^	93.8 ^a^	97.8 ^a^	36.3 ^a^	99.3 ^b^	81.7 ^a^	98.2 ^b^	68.6 ^a^	99.6 ^b^	98.4 ^a^	98.8 ^a^
Chloroquine	9.3 ^a^	82.1 ^b^	0.0 ^a^	4.0 ^b^	1.6 ^a^	95.5 ^b^	57.7 ^a^	91.9 ^b^	15.5 ^a^	61.0 ^b^	0.0 ^a^	21.7 ^b^
SP	51.2 ^a^	15.8 ^b^	66.9 ^a^	57.9 ^a^	22.0 ^a^	9.2 ^a^	62.7 ^a^	78.3 ^a^	49.9 ^a^	68.2 ^a^	65.5 ^a^	80.2 ^a^
Quinine	92.5 ^a^	24.0 ^b^	85.7 ^a^	84.3 ^a^	23.3 ^a^	10.7 ^a^	20.0 ^a^	9.2 ^a^	59.0 ^a^	82.9 ^b^	89.1 ^a^	16.8 ^b^
Quinine injection	60.0 ^a^	8.0 ^b^	65.6 ^a^	40.7 ^b^	20.9 ^a^	9.5 ^a^	19.0 ^a^	0.6 ^b^	56.4 ^a^	32.6 ^a^	51.2 ^a^	8.7 ^b^

Oral artemisinin monotherapy	3.8 ^a^	0.7 ^a^	10.2 ^a^	40.5 ^b^	0.0 ^a^	< 0.1 ^a^	4.1 ^a^	45.1 ^b^	1.3 ^a^	13.3 ^b^	1.1 ^a^	2.5 ^a^

¹Statistical comparisons conducted across outlet types, within countries. Statistical difference is labelled with a superscript, a or b. Proportions with different letters in their superscripts differ significantly from one another within countries (p < 0.05 for all tests).

Availability of different categories of anti-malarials in public health facilities was also calculated among all censused public health facilities (see Additional File 1 Table 1). Among all public health facilities (excluding community health workers), stockage rates of first-line quality-assured ACT at the time of survey were as follows: Benin, 81%; the DRC, 82%; Madagascar, 80%; Nigeria 43%; Uganda, 72% and Zambia, 85%. The stocking rate of SP among all outlets in the public sector, used in intermittent preventive treatment of pregnant women (IPTp), also varied (Benin, 50%; the DRC, 70%; Madagascar, 46%; Nigeria 63%; Uganda, 62% and Zambia, 59%). Quinine injection, the government-recommended treatment for severe malaria for persons who cannot take oral medication, ranged in public sector availability as well: Benin, 61%; the DRC, 66%; Madagascar, 43%; Nigeria 16%; Uganda, 73% and Zambia, 53%.

### Price

First-line quality-assured ACT was free at public sector outlets in four of the six countries. In Benin and the DRC, where public-sector patients pay for anti-malarials, the median prices were $1.29 (Inter Quartile Range [IQR]: $1.29, $1.29) and $0.52 (IQR: $0.00, $1.29) respectively for an adult equivalent treatment dose (see Additional File 2 Table 2).

Private sector first-line quality-assured ACT prices varied considerably between countries (Table 4). Their median price per AETD was highest in Zambia ($9.63; IQR: $3.01, $11.04), over $6 in Nigeria (IQR: $5.05, $6.74), $4.50 in Uganda (IQR: $2.49, $5.97), around $3 in Benin (IQR: $1.94, $5.77), under $2 in the DRC (IQR: $1.03, $3.61), and lowest in Madagascar ($0.14; IQR: $0.10, $0.57), where subsidized ACT was distributed through a social marketing campaign.

**Table 4:** Median price in US dollar (inter-quartile range) for an adult-equivalent treatment dose in the private sector (tablet formulation only), by anti-malarial type

	Median $ (IQR)		
	
	Most **Popular**¹	First-line quality-assured ACT	Oral Artemisinin Monotherapy
Benin^2^	0.65 ^a ^(0.43, 1.08) N = 462	3.24 ^b ^(1.94, 5.77) N = 216	8.10 (8.07, 10.45) N = 56
DRC	0.39 ^a ^(0.26, 0.52) N = 1, 258	1.86 ^b ^(1.03, 3.61) N = 252	3.23 (2.45, 4.13) N = 956
Madagascar	0.36 ^a ^(0.36, 0.36) N = 1, 847	0.14 ^b ^(0.10, 0.57) N = 302	(0 and 7.33) N = 2
Nigeria^3^	0.54 ^a ^(0.40, 0.81) N = 4, 061	6.40 ^b ^(5.05, 6.74) N = 372	3.24 (2.70, 3.77) N = 1, 438
Uganda^4^	0.50 ^a ^(0.30, 0.75) N = 653	4.48 ^b ^(2.49, 5.97) N = 81	9.55 (7.96, 11.94) N = 229
Zambia	0.40 ^a ^(0.30, 0.61) N = 261	9.63 ^b ^(3.01, 11.04) N = 83	6.74 (5.72, 6.74) N = 16

¹ The most popular ACT was chloroquine in Madagascar, and SP in all other countries. The most popular anti-malarial is based on volumes of anti-malarials sold or distributed in the last week, within each country.

^2 ^Statistical comparisons were conducted between the most popular treatment and the first-line quality-assured ACT. Statistical difference is labelled with superscripts 'a' or 'b'. Estimates with different letters in their superscripts differ significantly from one another within countries (p < 0.01 for all tests).

^3 ^In Nigeria, price is presented for the first-line quality-assured ACT (AL), rather than the alternate first-line quality-assured ACT (ASAQ). The median price for the alternate first-line treatment in Nigeria is $3.23 (1.89, 4.04) N = 622.

^4 ^In Uganda the sampled clusters included two areas located in districts that were undertaking a pilot of subsidized ACT in the retail sector [27]. Due to the presence of this pilot, the percent of private sector outlets stocking ACT was somewhat greater in these clusters than in the rest of the sample (50% and 19% respectively). These areas comprised 1.4% of the total sample of private outlets, and while accounting for only 5.4% of the private sector ACT products audited (25 out of 459 ACTs) they accounted for 17% of ACT products once sampling weights are taken into account (these clusters have high weights because they had a relatively low chance of selection under PPS). As in the pilot districts private sector ACT had a much lower price than elsewhere in the country, inclusion of the two pilot clusters can give a distorted picture of the average price available across the country as a whole. Uganda findings are therefore calculated both with and without the subsidized product observations from the 2 clusters for the first-line quality assured treatment. Data in the price table are presented excluding the subsidized product piloted in the clusters. The inclusion of the subsidized products provides a lower price for the first line quality assured treatment of $0.38 (0.38, 4.73) N = 104.

The median private sector AETD price of the most popular anti-malarial in tablet form, either SP or chloroquine in each country, ranged from $0.36 (IQR: $0.36, $0.36) to $0.65 (IQR: $0.43, $1.08). This was significantly lower (p < 0.01) than the median price of the first-line quality-assured ACT in each country, with Madagascar as an exception, where the first-line quality-assured ACT was significantly less expensive than the most popular anti-malarial, chloroquine.

Overall, the median price of chloroquine tablets ranged from $0.24 (IQR: $0.24, $0.48) to $0.48 (IQR: $0.48, $0.48) and SP from $0.38 (IQR: $0.29, $ 0.48) to $0.65 (IQR: $0.43, $1.08) in the private sector (see Additional File 3 Table 3).

Median prices of oral artemisinin monotherapies in the private sector ranged from $3.23 in the DRC (IQR: $2.45, $4.13) to $9.55 in Uganda (IQR: $7.96, $11.94).

### Market share

**Figure 2:**
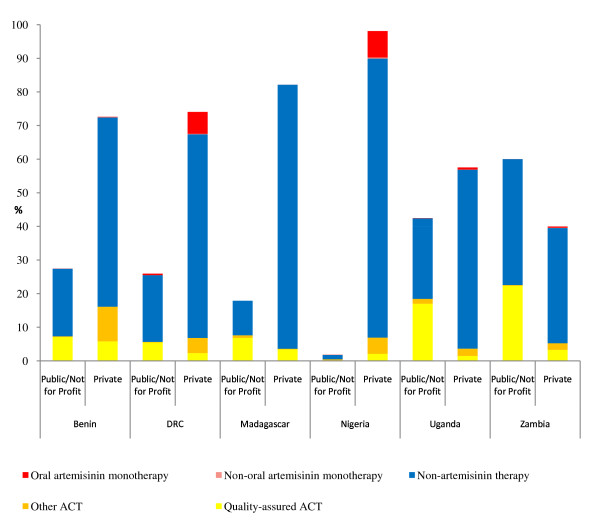
**Relative volumes of anti-malarials sold/distributed in the last seven days, by public/not-for-profit and private sectors and anti-malarial category¹**. ¹For each country, the public/not for profit and private columns sum to 100%. The figure shows 1) the contribution of the pubic/not for profit and private market share of each sector and 2) the breakdown of each sector's sales by antimalarial type.

Figure 2 shows the market share of different categories of anti-malarials sold or distributed in the seven days prior to the survey. With the exception of Zambia, the private sector played a larger role than the public/not-for-profit sector in the distribution of anti-malarials. This difference was most pronounced in Nigeria, where 98% of anti-malarial volumes were delivered through the private sector and only 2% of these were quality-assured ACT.

For most countries, non-artemisinin therapies dominated the overall market, followed by ACT and then oral artemisinin monotherapies. Exceptions to this finding were noted in Nigeria and the DRC where more oral artemisinin monotherapies were sold than ACT in the private sector. Across all countries quality-assured ACT accounted for less than 25% of total anti-malarial volumes; private-sector quality-assured ACT volumes represented less than 6% of the total market share. In the public/not-for-profit sector, SP made up the largest market share of non-artemisinin therapies (Benin, 11.6%; the DRC, 16.2%; Madagascar, 9.5%; Nigeria 1.4%; Uganda, 21.6%; Zambia, 34.1%).

### Provider knowledge

Knowledge of the first-line treatment was significantly higher in the public/not-for-profit sector than the private sector across all countries: 44% to 93% of providers in the public/not-for-profit sector could correctly state the first-line treatment, compared with 12% to 60% in the private sector (Table 5). Similar patterns were found for provider knowledge of the dosing regimens for the first-line treatment in children and adults. Knowledge of any dosing regimens was particularly low for the private sector in Benin and Madagascar, where less than 10% of providers were able to describe any child or adult regimen correctly.

**Table 5:** Provider knowledge of first-line treatment and dosing regimens, by sector¹

	Benin		DRC		Madagascar		Nigeria		Uganda		Zambia	
	
	Public/Not-for-Profit	Private	Public/Not- for-Profit	Private	Public/Not- for-Profit	Private	Public/Not- for-Profit	Private	Public/Not- for-Profit	Private	Public/Not- for-Profit	Private
	N=218	N=735	N=135	N=1, 242	N=575	N=2, 016	N=258	N=1, 839	N=563	N=699	N=178	N=283
Correctly state the recommended first-line treatment for uncomplicated malaria	73.0 ^a^	17.7 ^b^	76.4 ^a^	42.4 ^b^	71.7 ^a^	12.4 ^b^	43.7 ^a^	25.5 ^b^	92.8 ^a^	59.8 ^b^	92.7 ^a^	59.3 ^b^

Correctly state the dosing regimen of the first-line treatment for an adult	70.7 ^a^	8.4 ^b^	70.4 ^a^	28.5 ^b^	42.4 ^a^	5.1 ^a^	--^2^	--	86.6 ^a^	53.2 ^a^	92.7 ^a^	48.2 ^b^

Correctly state the dosing regimen of the first-line treatment for a two year old	70.2 ^a^	7.8 ^b^	69.6 ^a^	28.4 ^b^	68.8 ^a^	8.5 ^b^	--	--	89.5 ^a^	49.3 ^b^	92.7 ^a^	47.3 ^b^

¹ Statistical comparisons conducted across outlet types, within countries. Statistical difference is labelled with a superscript, a or b. Proportions with different letters in their superscripts differ significantly from one another within countries (p < 0.01 for all tests).

^2 ^Information on Nigeria's dosing regimen for their first-line alternative ACT, ASAQ, was not collected.

### Diagnostics

Each outlet was assessed for the availability of rapid diagnostic tests (RDTs) (see Additional File 4 Table 4). In the public/not-for-profit sector, availability varied considerably by country (Benin, 72%; the DRC, 36%; Madagascar, 43%; Nigeria, 7%; Uganda, 22% and Zambia, 86%). In the private sector, availability was less than 20% and significantly lower across all countries than in the public/not-for-profit sector.

## Discussion

This paper presents standardized, nationally representative data on the anti-malarial market in six sub-Saharan African countries between 2009 and 2010. The data confirm the low market share of ACT, reflecting a combination of low ACT availability in both the public/not-for-profit and private sectors, high ACT prices in the private sector and low provider ACT knowledge, particularly in the private sector. Other studies have highlighted similar challenges [8,10,11,17,28]. The following sub-sections describe the limitations of the *ACTwatch *outlet surveys, summarize the main findings according to availability, price, market share and provider knowledge, and address the policy implications of these results.

### Strengths and limitations

The *ACTwatch *survey methodology allows for the measurement of anti-malarial availability and prices in a standardized way, on a nationally representative sample of outlets, with multiple steps to ensure data quality. When combined with the *ACTwatch *household survey [29], and the supply chain study on the structure and operation of the anti-malarial distribution chain, *ACTwatch *data provide a full picture of the anti-malarial market, with multiple uses for policy making.

Despite the study's strengths, the outlet survey data presented here have several limitations. 1) Providers may have had incentives to mis-report certain information, such as whether they stocked any anti-malarials, or specific anti-malarial categories, especially if this was not permitted by government regulations. Although interviewers stressed that they were not connected with any regulatory body, anti-malarial availability and stocks may have been under-reported. 2) Volumes were based purely on provider recall. Given the lack of sales records in most private outlets, this was felt to be the most logistically feasible method for estimating volumes, but it is likely that some recall bias was present. 3) Adult equivalent treatment dose calculations were used when calculating prices and volumes. This has the advantage of allowing direct comparability between drugs. However, it should be noted that in practice many customers will be obtaining drugs for children and/or purchasing incomplete doses [30]. As a result, the price actually paid per customer may be considerably lower than the median price per AETD. Similarly, the actual number of customers to whom each drug was sold will be considerably higher than the volume of AETDs dispensed. It should also be noted that AETD prices presented in this paper do not include additional fees that may have been incurred for customers, such as consultation fees or service charges. 4) While price indicators were standardized according to the 2009 US dollar, it is acknowledged that there are different costs of living within countries and varying average or minimum wages, which make like-with-like comparisons across countries challenging. 5) Data were only collected at one point in the year, generally around the peak malaria transmission season. Seasonal variation may be expected, meaning in particular that anti-malarial availability and sales volumes may have been higher during the surveys than their annual averages, especially in countries with marked seasonal transmission, such as Madagascar and Zambia. 6) While a methodological strength of the survey is the full census of outlets in selected clusters, there are a number of practical implementation challenges with this method. Conducting a full census requires interviewers in the clusters to screen every outlet. It is acknowledged that some outlets with the potential to sell anti-malarials may have been missed, thus distorting the true figure of anti-malarial availability across countries.

### Availability

Overall availability of ACT was low, particularly in the private sector. In the private sector, non-artemisinin therapies, typically chloroquine or SP, were by far the most abundant anti-malarial available while first-line quality-assured ACT was available in less than one-quarter of the anti-malarial stocking outlets. Oral artemisinin monotherapies were available in more than 40% of private sector anti-malarial-stocking outlets in the DRC and Nigeria. Within the public/not-for-profit sector, quality-assured ACT stockage rates ranged between 49%-92%.

When observing stocking rates for all public health facilities, availability of ACT hovered around 80%, but in Nigeria less than half of facilities had a quality assured ACT in stock. This suggests that maintaining a consistent supply to public health facilities may be difficult, a finding supported by other facility based research, which also highlights stock-outs as a common problem in public health facilities [31].

### Price

With one notable exception in Madagascar, first-line quality-assured ACT treatment in the private sector was significantly more expensive, between 5-23 times so, than the most popular anti-malarial (chloroquine or SP). Given the importance of the private sector in distributing anti-malarials, the higher cost of first-line quality-assured ACT is a concern. Although ACT is free or at a low price in the public sector, this is not where the majority of people seek treatment [29].

In Madagascar, the price of quality-assured ACT in the private sector is exceptionally low at $0.14. This is due to the presence of a socially-marketed ACT, which is sold nationally at a highly subsidized price through pharmacies and drug stores. This mirrors findings from a pilot subsidy programme in Tanzania which demonstrated that after the introduction of an ACT subsidy, ACT prices were as low as $0.58 in the private sector and did not differ significantly from the price paid for SP, the most common alternative [32].

### Market share

Market share data highlight the dominant role of the private sector across all countries except Zambia, where 60% of all anti-malarials pass through the public/not-for-profit sector. Of the other countries, the private sector was the most important in Nigeria accounting for 98% of anti-malarial sales volumes, and elsewhere ranged from 58%-82%. Unfortunately, most of the anti-malarials sold through the private sector are non- artemisinin therapies, as well as concerning amounts of oral artemisinin monotherapies in the DRC and Nigeria (7%-8% of the total market). ACT comprised less than 25% of the total volume of anti-malarials distributed across all sectors. Quality-assured ACT fared even worse in the private sector, where it generally accounted for less than 6% of the total market share.

Of note, in Nigeria and the DRC, the market share of ACT was particularly low and more oral artemisinin monotherapies were sold than ACT in the private sector. In Nigeria, oral artemisinin monotherapy is less expensive than first-line quality-assured ACT treatment, perhaps explaining why higher volumes of oral artemisinin monotherapies are found.

### Provider knowledge

Provider knowledge was generally lower in the private sector than in the public/not- for-profit sector. Other studies have shown that medicine seller knowledge of drugs and doses, particularly in the private sector, is often poor [13,15,16]. This is likely to be exacerbated by the relatively recent introduction of ACT in sub-Saharan Africa because these have new dosage requirements and more than one product may be available with different dosages [13].

### Policy implications

Poor ACT availability in both public/not-for-profit and private sectors, as well as the high price observed in the private sector, and poor provider knowledge, serve as barriers to obtaining effective treatment. Of grave concern, quality-assured ACT availability in the public sector ranges between 43-85%. This suggests that there are issues within the public-sector supply chain that require urgent attention, especially in Nigeria and Uganda where stocking rates were lowest.

In the private sector, non-artemisinin therapies are more widely available and more commonly sold than other classes of anti-malarials; these medicines are also usually sold at a much lower price than ACT. Given the relative affordability and accessibility of non-artemisinin therapies, consumers have strong incentives to choose the 'wrong', ineffective, anti-malarial when seeking treatment.

Oral artemisinin monotherapy, discouraged for its potential to speed the spread of artemisinin resistance, was commonly available and sold at private outlets in the DRC and Nigeria. These disappointing findings should act as a rallying call given the Millennium Development Goal targets and that together these two nations account for nearly half of the total global burden of malaria disease [33]. While all the *ACTwatch *countries have taken regulatory measures to withdraw the marketing authorization of oral artemisinin monotherapies, the manufacturing and marketing of these products is still ongoing (and many of these companies are located in Nigeria, though renewal licenses will not be granted to existing companies currently marketing oral artemisinin monotherapies [34]). This calls into question the effectiveness of current regulatory practices in countries where this drug is banned. Interventions such as tighter regulation and building awareness of issues with oral artemisinin monotherapies should be considered so that this national policy of public health significance is indeed followed. For other countries, availability of oral artemisinin monotherapy was low, suggesting a positive outcome from the 2006 WHO regulatory ban on oral artemisinin monotherapy, and heightened government response to the threat of artemisinin resistence.

As the majority of anti-malarials are delivered through the private sector, there is a clear need to consider how to handle the role of the private sector when developing anti-malarial drug policies. Some argue that emphasis should remain on the public sector, to attract more people to outlets where care is regulated and provided by qualified staff [35]. Others argue that, given the current state of developing country health systems, while the public sector cannot be ignored, high private sector use is inevitable in many countries, and therefore strategies should be adopted to improve its quality [36]. The extent to which the emphasis should be placed on the private sector continues to be debated [37] and data presented here provide evidence to help facilitate further discussion.

Prominent among private sector initiatives is the Affordable Medicines Facility for Malaria (AMFm), which began operations in pilot countries during 2010, and provides a mechanism for heavily subsidized, quality-assured ACT to be sold at a target retail price of $0.50 [21]. However, there continues to be controversy about such initiatives, with some arguing that the subsidy will be captured by middle-men in the distribution chain; that market power in the private sector will lead to continued problems of affordability and inaccessibility by the poor; and that widespread availability of ACT without improving access to diagnosis will result in mis-use of ACT and fuel the development of resistance [35,38].

Of the countries covered in this paper, Madagascar provides an interesting example of a setting where an ACT subsidy has been in operation since 2008. The subsidized ACT was open to all wholesalers registered or unregistered in the country and it was sold nationally through community health workers, pharmacies and rural drug stores. The ACT did not have over-the-counter status and had a recommended retail price of $0.05. In contrast to the other countries, the price of quality-assured ACT was lower than non-artemisinin therapies. However, accessibility remained low, as ACTwatch data show that less than 10% of private sector outlets stocking this type of ACT. Provider knowledge of the first-line treatment was also low (12%). As such, substantial gains in market share of ACT were not apparent, and rates of ACT use in febrile children remained less than 5% [29]. Despite this, smaller scale pilots of ACT subsidies have shown impressive results [27,32,39]. Promising findings have also been demonstrated in Cambodia, where an ACT social marketing programme has been in place for the past 10 years [40].

The data suggest a need for interventions to address a full range of supply side issues that go above and beyond a price subsidy (i.e. targeting provider knowledge, increasing supply and product promotion). Interventions should also focus on demand creation through activities that include behaviour change communication, to encourage the stocking and uptake of ACT, particularly in the private sector. It remains to be seen how far the "supporting interventions, " (e.g. provider trainings and behaviour change communication, all to promote ACT utilization), being implemented as part of the AMFm and other initiatives will go in addressing these concerns.

## Conclusions

*ACTwatch *provides standardized, nationally representative data on anti-malarial supply from multiple countries collected within 16 months. Data presented in this paper report on key, policy relevant indicators across countries. The results typically demonstrate the low availability, low market share and high price of ACT, at least in the private sector where most anti-malarials are being accessed, with some exceptions. Data from *ACTwatch *outlet surveys confirm that there is substantial room to improve availability and affordability of ACT treatment in six countries across sub-Saharan Africa, and in both the public/not-for-profit and private sectors. The data will also be useful for monitoring the impact of interventions such as the Affordable Medicines Facility for malaria. More detailed, country specific findings (for example, outlet anti-malarial availability by country strata) can be found in country reports on the *ACTwatch *website [41].

## Competing interests

The authors declare that they have no competing interests.

## Authors' contributions

KOC, SC, DC devised the study design and objectives. CBA, JAA, BFR, BC, DR, BH, CZ, LA, JR, EA, PM, FM, TS contributed to the country specific study design adaptations and interpretations. CZ, LA, JR, EA, PM, FM, TS, EM, JN contributed to field implementation and data collection. HG, JN, IE, EM, SP, KOC contributed to the analysis. KOC wrote the first draft of the manuscript. DC, TS, CG, KH, HG, EM, SP contributed to interpretation and subsequent drafts of the manuscript. All authors read and approved the final manuscript.

## Supplementary Material

Additional file 1 **Proportion of all public health facilities stocking at least one anti-malarial on the day of survey**. This table shows the availability of different categories of anti-malarials in public health facilities as the proportion of facilities with anti-malarials in stock, among all censused public health facilities. Click here for file

Additional file 2 **Median price in US dollar (inter-quartile range) of adult-equivalent anti-malarial treatment doses in the public sector (tablet formulation)**. This table shows the median price of the first-line quality assured ACT anti-malarial treatment doses in the public sector across all countries. Click here for file

Additional file 3 **Median price in US dollar (inter-quartile range) of adult-equivalent anti-malarial treatment doses in the private sector (tablet formulation), by antimalarial type**. this table shows the median price of SP and CQ anti-malarial treatment doses in the private sector across all countries. Click here for file

Additional file 4 **Percent of outlets stocking RDTs, by outlet category**. This table shows the availability of RDTs in public/not for profit and private sectors across all countries. Click here for file
