# Supplementary material for: Got ACTs? Availability, price, market share and provider knowledge of anti-malarial medicines in public and private sector outlets in six malaria-endemic countries
Source: Malar J. 2011 Oct 31;10:326. doi: 10.1186/1475-2875-10-326 (PMC3227612; doi:10.1186/1475-2875-10-326)
Supplement: Additional file 1 — Proportion of all public health facilities stocking at least one anti-malarial on the day of survey. This table shows the availability of different categories of anti-malarials in public health facilities as the proportion of facilities with anti-malarials in stock, among all censused public health facilities. [file 1475-2875-10-326-S1.DOC]

Additional File 1: Proportion of all public health facilities stocking at least one anti-malarial on the day of survey

|  |  | **Benin** | **DRC** | **Madagascar** | **Nigeria** | **Uganda** | **Zambia** |
| --- | --- | --- | --- | --- | --- | --- | --- |
|  | | N=182 | N=111 | N=531 | N=255 | N=525 | N=165 |
| Any ACT | | 81.8 | 85.3 | 81.1 | 55.8 | 73.8 | 85.3 |
| First-line quality assured ACT | | 80.5 | 81.7 | 80.4 | 42.5 | 72.1 | 85.2 |
| Quality assured ACT | | 80.5 | 81.7 | 80.4 | 42.5 | 72.1 | 85.2 |
| Any non-artemisinin therapy | | 90.8 | 90.4 | 73.8 | 80.6 | 88.0 | 95.7 |
| Chloroquine | | 1.4 | - | 2.0 | 63.0 | 20.7 | - |
| SP | | 49.7 | 69.5 | 45.5 | 63.4 | 61.5 | 58.5 |
| Quinine | | 85.9 | 86.0 | 48.2 | 17.2 | 75.2 | 91.4 |
| Quinine injection | | 60.7 | 66.3 | 43.2 | 16.0 | 72.9 | 52.9 |
| Oral artemisinin monotherapy | | 1.1 | 10.3 | 0.0 | 4.3 | 0.2 | 1.3 |
