# Supplementary material for: Got ACTs? Availability, price, market share and provider knowledge of anti-malarial medicines in public and private sector outlets in six malaria-endemic countries
Source: Malar J. 2011 Oct 31;10:326. doi: 10.1186/1475-2875-10-326 (PMC3227612; doi:10.1186/1475-2875-10-326)
Supplement: Additional file 2 — Median price in US dollar (inter-quartile range) of adult-equivalent anti-malarial treatment doses in the public sector (tablet formulation). This table shows the median price of the first-line quality assured ACT anti-malarial treatment doses in the public sector across all countries. [file 1475-2875-10-326-S2.DOC]

Additional File 2: Median price in USD (inter-quartile range) of adult-equivalent anti-malarial treatment doses in the public sector (tablet formulation)

|  |  |
| --- | --- |
|  | **First-line quality assured ACT** |
| Benin | 1.29 (1.29, 1.29) N = 473 |
| DRC | 0.52 (0.00, 1.29) N = 184 |
| Madagascar | 0.00 (0.00, 0.10) N = 1,396 |
| Nigeria | 0.00 (0.00, 0.00) N = 55 |
| Uganda | 0.00 (0.00, 0.00) N = 693 |
| Zambia | 0.00 (0.00, 0.00) N = 508 |
