# Supplementary material for: Got ACTs? Availability, price, market share and provider knowledge of anti-malarial medicines in public and private sector outlets in six malaria-endemic countries
Source: Malar J. 2011 Oct 31;10:326. doi: 10.1186/1475-2875-10-326 (PMC3227612; doi:10.1186/1475-2875-10-326)
Supplement: Additional file 3 — Median price in US dollar (inter-quartile range) of adult-equivalent anti-malarial treatment doses in the private sector (tablet formulation), by antimalarial type. this table shows the median price of SP and CQ anti-malarial treatment doses in the private sector across all countries. [file 1475-2875-10-326-S3.DOC]

Additional File 3: Median price in USD (inter-quartile range) of adult-equivalent anti-malarial treatment doses in the private sector (tablet formulation), by antimalarial type

|  |  |  |
| --- | --- | --- |
|  | **CQ** | **SP** |
| Benin | 0.32 (0.24, 0.41) N = 346 | 0.65 (0.43,1.08) N = 462 |
| DRC | 0.26 (0.26, 0.39) N = 10 | 0.39 (0.26, 0.52) N = 1,258 |
| Madagascar | 0.36 (0.36, 0.36) N = 1,847 | 0.38 (0.29, 0.48) N = 477 |
| Nigeria | 0.39 (0.20, 0.67) N = 1,159 | 0.54 (0.40, 0.81) N = 4,061 |
| Uganda | 0.24 (0.24, 0.48) N = 282 | 0.50 (0.30, 0.75) N = 653 |
| Zambia | 0.48 (0.48, 0.48) N = 55 | 0.40 (0.30, 0.61) N = 261 |
