# Supplementary material for: Got ACTs? Availability, price, market share and provider knowledge of anti-malarial medicines in public and private sector outlets in six malaria-endemic countries
Source: Malar J. 2011 Oct 31;10:326. doi: 10.1186/1475-2875-10-326 (PMC3227612; doi:10.1186/1475-2875-10-326)
Supplement: Additional file 4 — Percent of outlets stocking RDTs, by outlet category. This table shows the availability of RDTs in public/not for profit and private sectors across all countries. [file 1475-2875-10-326-S4.DOC]

Additional File 4. Percent of outlets stocking RDTs, by outlet category

|  |  | **Benin** | | **DRC** | | **Madagascar** | | **Nigeria** | | **Uganda** | | **Zambia** | |
| --- | --- | --- | --- | --- | --- | --- | --- | --- | --- | --- | --- | --- | --- |
|  |  | Public/Not- for-Profit | Private | Public/Not- for-Profit | Private | Public/Not- for-Profit | Private | Public/Not- for-Profit | Private | Public/Not- for-Profit | Private | Public/Not- for-Profit | Private |
|  | | N=220 | N=812 | N=132 | N=  1,239 | N=575 | N=2,020 | N=258 | N=  1,824 | N=560 | N=689 | N=178 | N=279 |
| RDTs | | 71.9 ^a^ | 0.8 ^b^ | 36.4 ^a^ | 6.8 ^b^ | 43.0 ^a^ | 0.4 ^b^ | 6.8 ^a^ | 0.7 ^b^ | 21.5 ^a^ | 9.0 ^b^ | 85.7 ^a^ | 16.9 ^b^ |
